# Reply to Comment on Lecca, L.I.; Portoghese, I.; Mucci, N.; Galletta, M.; Meloni, F.; Pilia, I.; Marcias, G.; Fabbri, D.; Fostinelli, J.; Lucchini, R.G.; Cocco, P.; Campagna, M. Association between Work-Related Stress and QT Prolongation in Male Workers

**DOI:** 10.3390/ijerph17020510

**Published:** 2020-01-14

**Authors:** Luigi Isaia Lecca, Igor Portoghese, Nicola Mucci, Maura Galletta, Federico Meloni, Ilaria Pilia, Gabriele Marcias, Daniele Fabbri, Jacopo Fostinelli, Roberto G. Lucchini, Pierluigi Cocco, Marcello Campagna

**Affiliations:** 1 Department of Experimental and Clinical Medicine, University of Florence Largo Piero Palagi 1, 50139 Florence, Italy; nicola.mucci@unifi.it; 2 Department of Medical Sciences and Public Health, University of Cagliari, Blocco I, SS 554, km 4,500, 09042 Monserrato, Italy; igor.portoghese@gmail.com (I.P.); maura.galletta@gmail.com (M.G.); federicomeloni@hotmail.it (F.M.); cromatinap@gmail.com (I.P.); gabriele.marcias@libero.it (G.M.); daniele.fabbri@hotmail.it (D.F.); pcocco@unica.it (P.C.); mam.campagna@gmail.com (M.C.); 3 Department of Civil and Environmental Engineering and Architecture, University of Cagliari, 09123 Cagliari, Italy; 4 Occupational Medicine, University of Brescia, P.le Spedali Civili 1, 25123 Brescia, Italy; jacopo.fostinelli@unibs.it (J.F.); roberto.lucchini@unibs.it (R.G.L.); 5 Division of Occupational and Preventive Medicine, Icahn School of Medicine at Mount Sinai, New York, NY 10029, USA

We appreciate the interest raised by our paper on the association between conditions linked to work-related stress and the prolongation of the QT interval on the electrocardiogram [1]. Alizargar et al., in their commentary [2], raise two main concerns, namely: (1) the operational location would be the main determinant of QT interval prolongation, and (2) one would have to assess Pearson’s correlation “before analyzing the assumptions that should be made before such an analysis”. Alizargar et al. [2] state that “if both variables are normally distributed a Pearson’s correlation [is appropriate] and if the normality assumption is not met, a Spearman’s correlation analysis is appropriate”.

As for the first point, Alizargar et al.’s concern refers to the results of our multivariate analysis, which showed that, in spite of the significant, although weak, correlation we observed in the univariate analysis between age and the frequency corrected QT interval (QTc) and the QT index (QTi) having been slightly weakened in the multiple regression model, we stated: “Age was a clear contributing factor in QTc and QTi prolongation in our study. Consistent with other studies, this finding supports the influence of aging on cardiovascular parameters. This aspect is of special interest, considering the constant, progressive aging of the working population in various lines of work, which elevates the importance of assessing inexpensive, and simple-to-apply early biomarkers, such as the QT parameters, to predict adverse CV outcomes”.

In the opinion of Alizargar et al., “This statement is wrong as the effects of age is the result of operating location and physical activity, but as there is a borderline *p*-value of 0.048 and 0.047 in the site 1 and 2, respectively, between physical activity and QTc, we can assume that the main confounder can be considered the site location. Unfortunately, they did not include all the risk factors like duration of employment in the multivariate analysis; hence the conclusion about the main risk factor affecting QTc is questionable”. The foundation of their opinion is that “the observed effect of age in the correlation analysis disappears when they controlled for some factors”.

Instead, in our paper, we did not discard the effect of aging, as it was repeatedly reported in the literature [3,4], and, far from disappearing, it was still directly associated with length of the QT interval, although the regression coefficient was no longer significant. We also stated that “a combination of organizational factors (workload scheduling, shiftwork modality, and job support), personal factors (age, physical activity, educational level), and other factors we could not assess in our study might contribute to modifying specific cardiac outcomes”.

The scope of the correlation matrix (Table 2 in our paper) was to find out what covariates correlated with QTc and QTi, so as to select those to be included in the multiple regression model, reciprocally adjusting for their individual effect. The duration of employment was not associated with the QT interval parameters, but was strongly associated with age, weakly directly associated with heart rate and job control, and inversely associated with education and shiftwork. Therefore, we did not include the duration of employment as a covariate in the regression model, because there was collinearity with age, and no association with the QT parameters.

As for the second point, in our paper, we did not show the results of the tests for the assumption of normality that we conducted on the continuous variables before testing their correlation, because we thought it would have required further text, while not contributing additional, meaningful information. 

Several assumptions need to be matched when considering what type of test, whether parametric or nonparametric, has to be conducted when testing the correlation between two variables—the variables need to be continuous; each individual needs to have a pair of values; the absence of outliers (±3.29 standard deviations from the mean); and the linearity, homoskedasticity, and approximate normality of the distribution of both variables. An exact normality is unnecessary, but at least one of the two variables must have a normal distribution, unless a valid confidence interval of the regression coefficient has to be calculated [5].

We clearly stated in the main text and in Table 2 that “We assessed the correlation between parametric and nonparametric variables with the Pearson’s or Spearman’s correlation coefficient, as appropriate”. As we stated regarding the first point, the idea of the correlation matrix was to select the covariates to be included in a multiple regression model from those showing a significant correlation with QTc and QTi, so as to reciprocally adjust for their individual effect. The variables were continuous or discrete, and according to their type, we applied the appropriate correlation test. To answer to Alizargar et al.’s commentary, Table 1 shows the results of the Kolmogorov–Smirnov test, and the associated *p*-value for each of the continuous variables (correlation matrix).

As shown in Table 1, the distribution of several variables departed significantly from normality. However, only for the duration of employment, was there an important difference between the mean and median values, and the maximum value was more than 3.29 *sd* far from the mean value for only the duration of employment and the heart rate. Still, using either the Pearson correlation or the Spearman correlation analysis, the duration of employment did not show an association with QTc and QTi, but only with age and heart rate; in all of the other instances, as shown in Table 2 of our paper (1), we used the Spearman correlation analysis. The heart rate was the correction factor in calculating QTc and QTi, and was therefore collinear with both metrics. The systolic and diastolic blood pressure were also collinear. We previously explained why we did not include duration of employment as a covariate.

In criticizing our conclusive statements, Alizargar et al. speculate that “workers with subclinical cardiovascular disease might choose not to take long and night shifts. So, the fact that site 2 workers had prolonged QTc interval might be because they are healthier than site 1 workers and not because the long and night shift working, caused [a] prolong[ed] QTc interval”. 

Readers familiar with the topic would be well aware that a prolonged QT interval is the early adverse outcome we aimed to investigate as an indicator of a work-related stressful condition, and not an indicator of a healthier condition, as Alizargar et al. seem to suggest. As we explained in the main text, the difference between the two operational sites was related to the work organization, namely: scheduled high activity periods followed by low activity in Site 1 vs. a more regular work activity in site 2; also, as shown in Table 1, site 2 workers were more frequently engaged in 12-h shifts, including night shifts, but the same circumstance also concerned 11% of workers in Site 1. Therefore, if any, a subclinical cardiovascular disease would have more likely implied changing the job assignment within the same operational location, rather than changing the location. A subclinical alteration is by definition that which cannot reach the individual perception of disease; detecting it with a proper biomarker has a preventive value, and was the scope of our paper. To minimize the possible bias related to the possible differential distribution of subjects with a clinical manifestation of disease by location, job, and/or type of work-shift schedule, and for the possible association between their disease and a prolonged QT interval, we excluded 60 subjects from study with a “*prior diagnosis of any cardiovascular disease (ischemic heart disease, arrhythmia, cardiac hypertrophy, hypertension), diabetes, any neoplastic disease, and* [those] *being under medication with drugs potentially causing a prolonged QT interval*” [1]. Finally, we presented the results of an observational cross-sectional study and an unplanned experimental scenario, and therefore, the differences in mean age and duration of employment were real situations. We are confident our analysis properly managed the challenges posed by the study design.

## Figures and Tables

**Table 1:** Test for the assumption of normality of the distribution of the continuous variables presented in Table 2 of Lecca et al. (1). BMI—body mass index; KS—Kolmogorov–Smirnov; SD—standard deviation.

Variables	KS Test D-Value	*p*-Value	Mean, SD	Median (Range)
Age	0.066	0.1376	42, 7.54	43 (20–63)
Duration of employment	0.186	<0.00001	7, 7.43	4 (1–39)
BMI	0.095	0.0086	25, 2.51	25 (19–34)
Systolic blood pressure	0.133	0.00004	124, 12.24	125 (80–170)
Diastolic blood pressure	0.201	<0.00001	79, 7.73	80 (50–105)
Heart rate	0.082	0.0342	65, 11.65	65 (39–132)
QTc	0.038	0.7607	401, 25.69	402 (322–491)
QTi	0.064	0.1654	97, 5.58	97 (81–116)
